# Syndromic Multiple Odontogenic Keratocysts Associated With Impacted Teeth: A Rare Case Report

**DOI:** 10.1155/crid/9907993

**Published:** 2026-09-17

**Authors:** Faizal Manrapi, Mohammad Gazali, Nurwahida, Nisrina Ekayani Nasrun

**Affiliations:** ^1^ Department of Oral and Maxillofacial Surgery, Faculty of Dentistry, Hasanuddin University, Makassar, Indonesia, unhas.ac.id; ^2^ Dental Hospital of Hasanuddin University, Makassar, Indonesia

**Keywords:** case report, Gorlin–Goltz syndrome, impacted teeth, jaw cysts, odontogenic keratocyst

## Abstract

**Introduction:**

Odontogenic keratocysts are developmental odontogenic cysts that may occur as solitary lesions or as multiple jaw lesions. Multiple odontogenic keratocysts in young patients should raise suspicion of an underlying syndrome, particularly Gorlin–Goltz syndrome.

**Case Presentation:**

A 15‐year‐old male was referred to the Dental Hospital of Hasanuddin University after a 9‐month history of progressive left mandibular enlargement. Clinical and radiographic assessment revealed multiple cystic lesions involving the maxilla and mandible, with impacted teeth in several regions. Chest radiography showed bifid left ribs, and computed tomography showed bilateral calcification of the falx cerebri. Histopathological examination confirmed odontogenic keratocystic lesions.

**Therapeutic Intervention and Outcomes:**

The patient underwent staged enucleation of multiple maxillary and mandibular cysts under general anesthesia. The postoperative course was uneventful. Obturator rehabilitation was provided, and follow‐up radiographic evaluation showed early bone formation. The final diagnosis was syndromic multiple odontogenic keratocysts of the maxilla and mandible associated with Gorlin–Goltz syndrome.

**Conclusion:**

Multiple odontogenic keratocysts in adolescents require careful syndromic evaluation. Early diagnosis, staged surgical management, long‐term surveillance, and multidisciplinary collaboration are essential to reduce recurrence and identify associated systemic manifestations.

## 1. Introduction

Odontogenic cysts are among the most frequent cystic lesions of the jaws. Odontogenic keratocyst (OKC) is a developmental odontogenic cyst that arises from remnants of the dental lamina and is clinically important because of its growth pattern and tendency to recur [1] Although most odontogenic cysts present as single jaw lesions, multiple OKCs in a young patient are unusual and should prompt assessment for Gorlin–Goltz syndrome, also known as nevoid basal cell carcinoma syndrome [1].

Syndromic OKCs differ from isolated lesions because they may be multiple, bilateral, recurrent, and associated with extraoral findings. Previous reports describe multiple OKCs with or without syndromic features and emphasize the need for radiographic evaluation, histopathological confirmation, and long‐term follow‐up [2–5]. Treatment selection depends on lesion size, anatomical extension, relationship to impacted teeth, and risk of recurrence. Enucleation is commonly used for smaller or accessible lesions, whereas marsupialization, decompression, adjunctive chemical cauterization, or resection may be considered for extensive or recurrent lesions [2–4].

This report describes a rare adolescent case of syndromic multiple OKCs associated with impacted teeth and radiologic features consistent with Gorlin–Goltz syndrome. The report highlights diagnostic reasoning, staged surgical treatment, rehabilitation, and follow‐up considerations.

## 2. Case Report

A 15‐year‐old male was first seen at Lagaligo Hospital, East Luwu, with enlargement of the left mandible that had progressed for approximately 9 months. One year before referral, he had recurrent pain in the posterior left mandibular teeth. Four months later, he noticed a marble‐sized swelling that gradually enlarged. In February 2025, an oral surgeon at Lagaligo General Hospital performed an incisional biopsy of the left mandibular lesion. The patient was then referred to the Department of Oral and Maxillofacial Surgery, Dental Hospital of Hasanuddin University, Makassar, for definitive management. In March 2025, he reported intraoral discharge with a salty taste and foul odor. No family history of a similar condition was reported.

**Figure 1:**
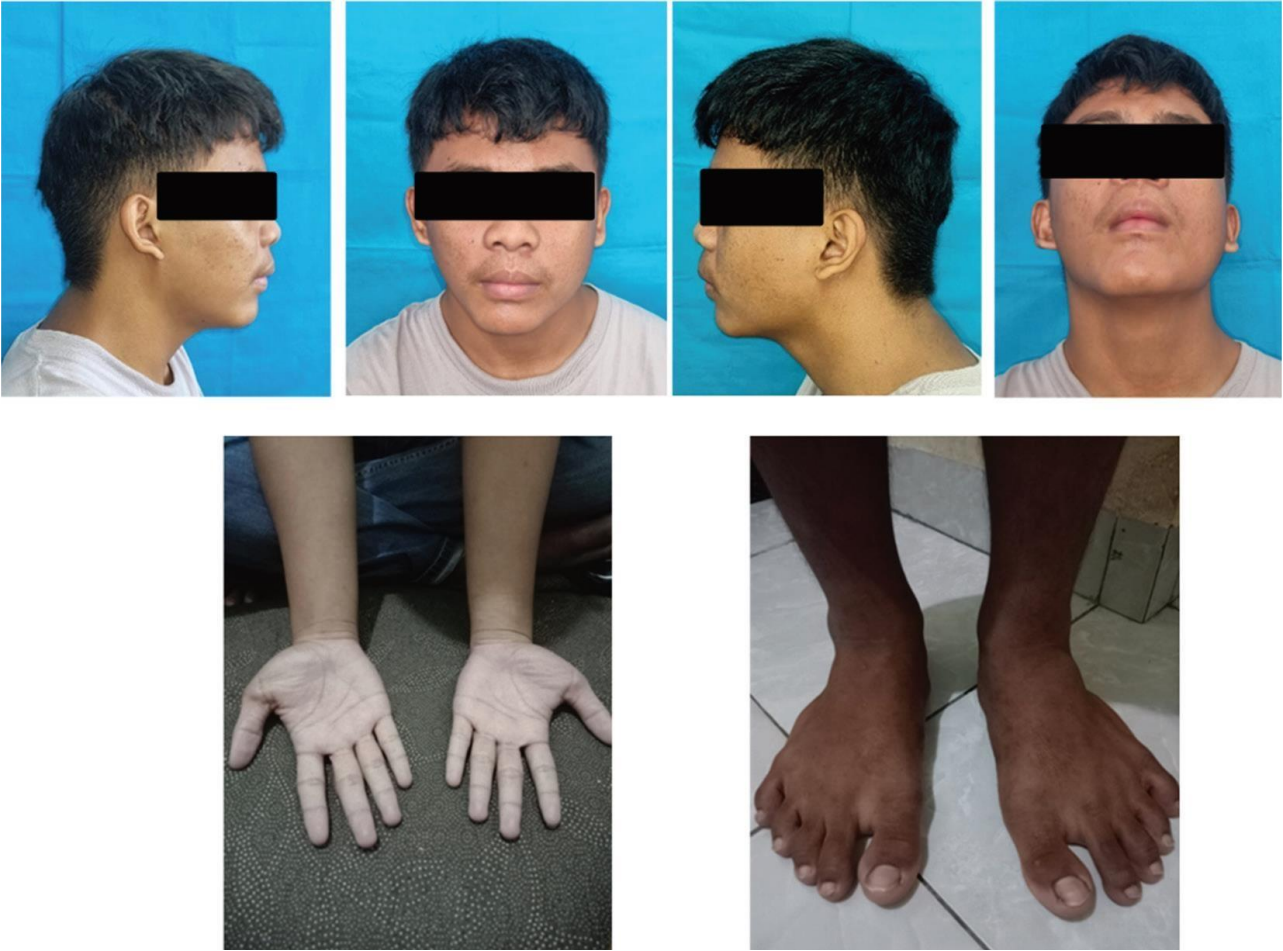
Extraoral view showing facial asymmetry (top image) and plantar and palmar views of the patient (bottom image).

**Figure 2:**
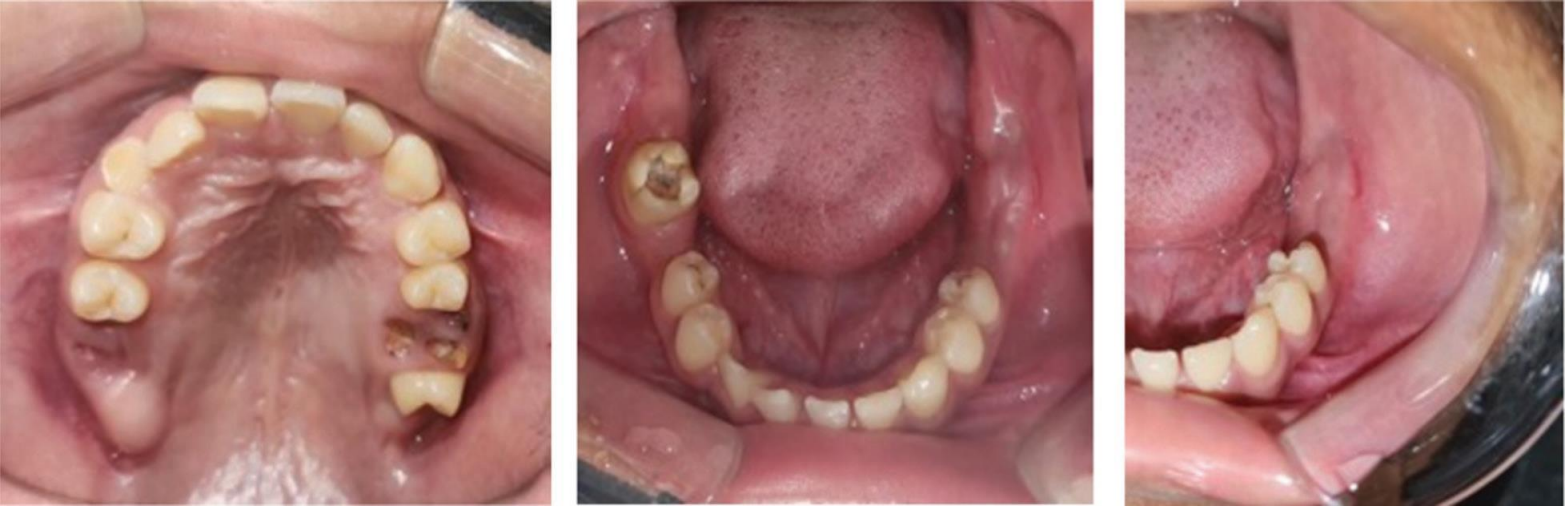
Intraoral view showing enlargement in the edentulous regions of Teeth 36–38 and 26–28.

Extraoral examination showed facial asymmetry caused by swelling in the left posterior mandibular region (Figure 1). Intraoral examination identified enlargements in the edentulous areas of Teeth 37–36, the anterior mandibular region of Tooth 43, the anterior maxillary region of Tooth 13, the maxillary region of Teeth 27–28, and the posterior region of Tooth 48 (Figure 2).

**Figure 3:**
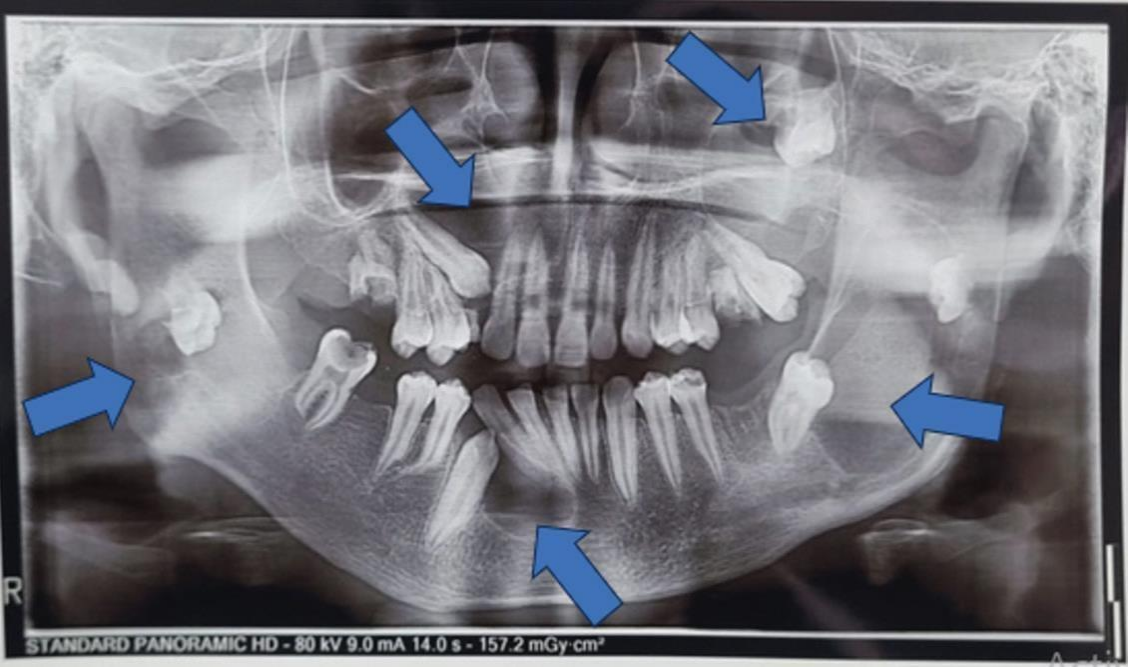
Panoramic radiograph showing multiple cystic lesions in Regions 13, 28, 38, 43, and 48.

**Figure 4:**
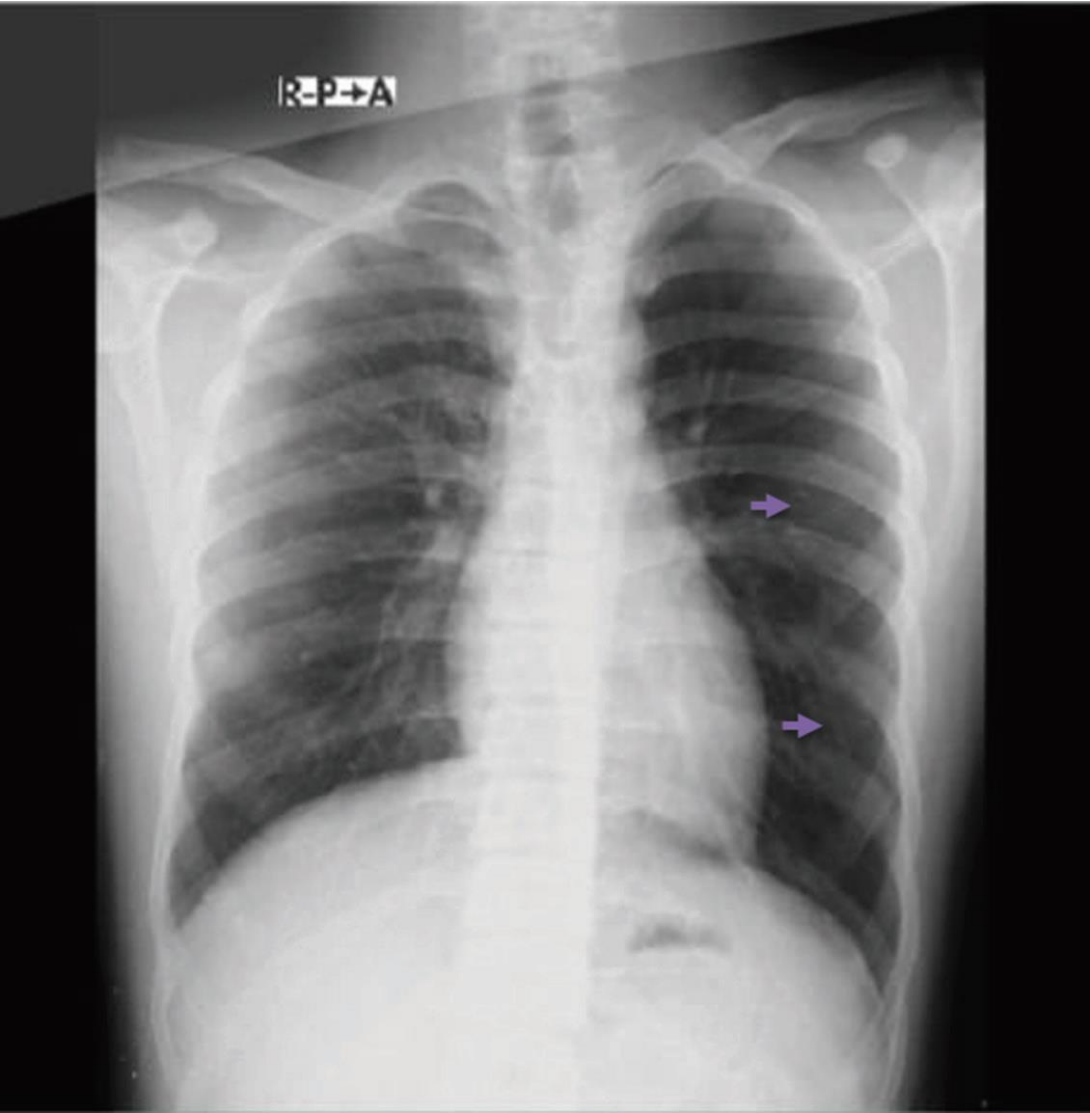
Chest X‐ray showing bifid left costae at the third and fifth ribs.

**Figure 5:**
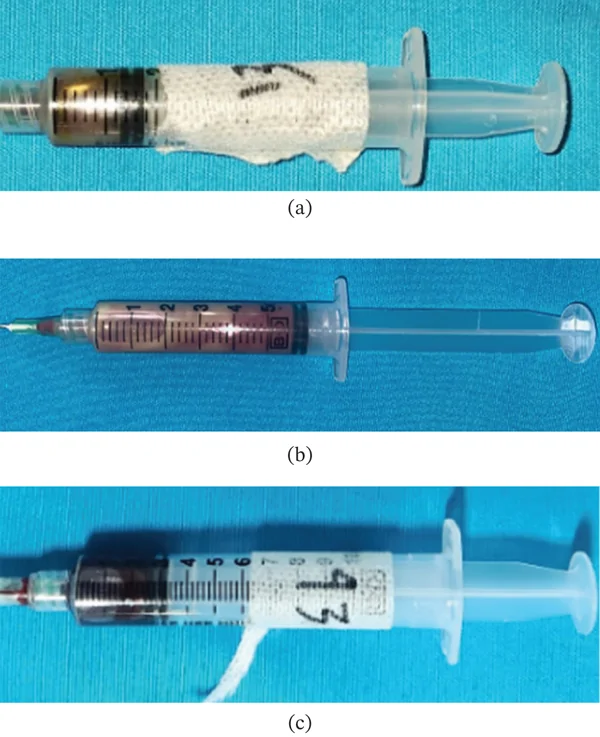
Aspiration test findings. (a) Region 13. (b) Region 43. (c) Region 38.

**Figure 6:**
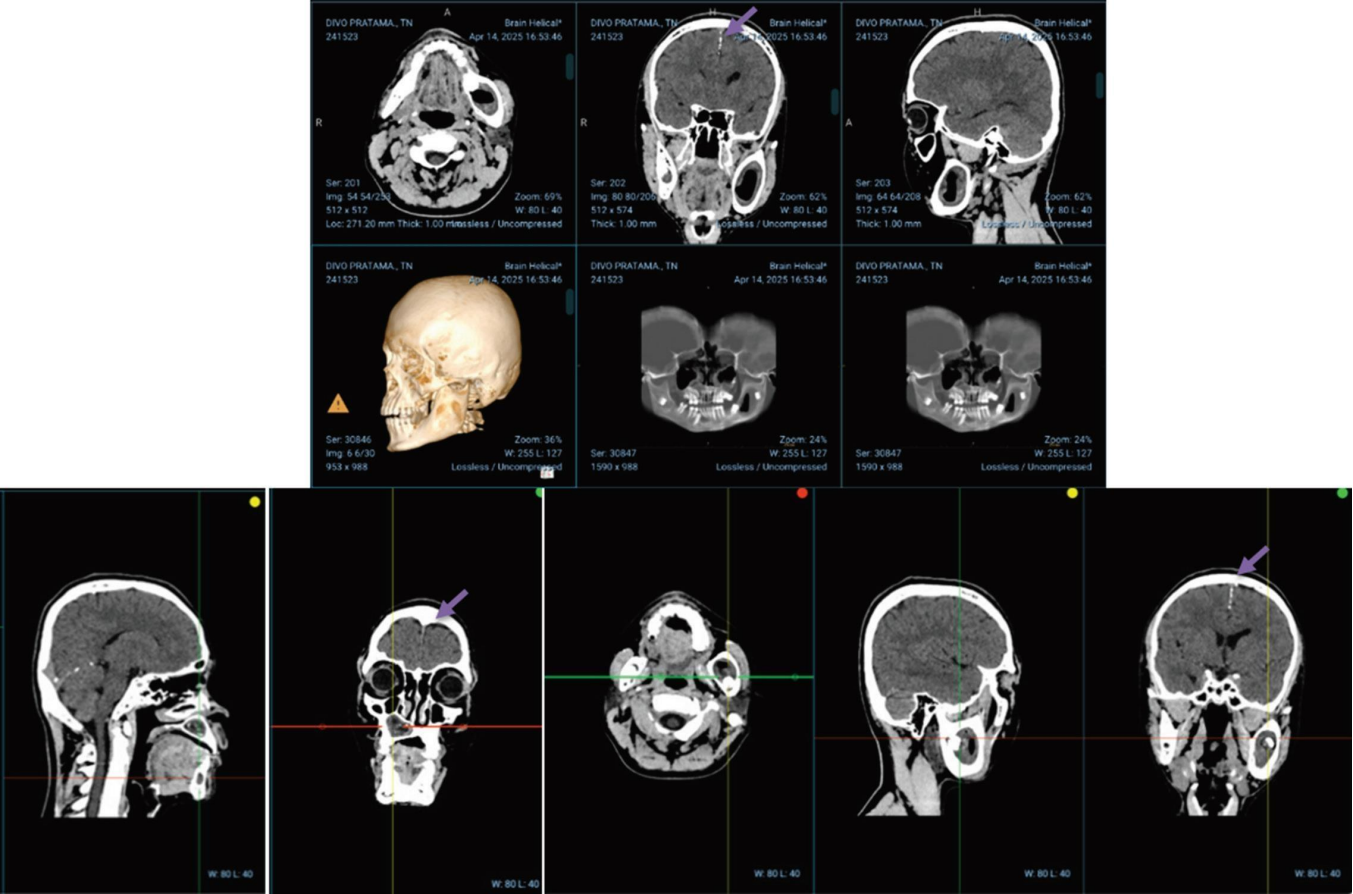
CT scan showing bilateral calcification of the falx cerebri.

Laboratory findings were within normal limits. Panoramic radiography demonstrated multiple radiolucent jaw lesions involving the maxilla and mandible. Teeth 23, 24, and 25 were displaced superiorly, and Tooth 23 was rotated almost 180° toward the orbital floor. Two radiolucent areas were observed in the region of Tooth 33 and distal to Tooth 37; both had progressive expansion and well‐defined wavy borders (Figure 3). Chest radiography revealed bifid left costae at the third and fifth ribs (Figure 4). Aspiration showed pus mixed with blood. Region 43 yielded dark red fluid, and Region 13 yielded clear yellow fluid mixed with blood (Figure 5). Computed tomography showed bilateral calcification of the falx cerebri (Figure 6).

Before surgery, the patient and guardian received an explanation of the diagnosis, proposed treatment, possible complications, prognosis, and follow‐up requirements. Written informed consent was obtained. The patient underwent staged enucleation of multiple maxillary and mandibular cysts under general anesthesia with right nasotracheal intubation in the supine position. Intravenous ceftriaxone 1 g was administered as broad‐spectrum antibiotic prophylaxis. The left mandibular lesion was treated first because it had the highest risk of complications, particularly pathologic fracture.

**Figure 7:**
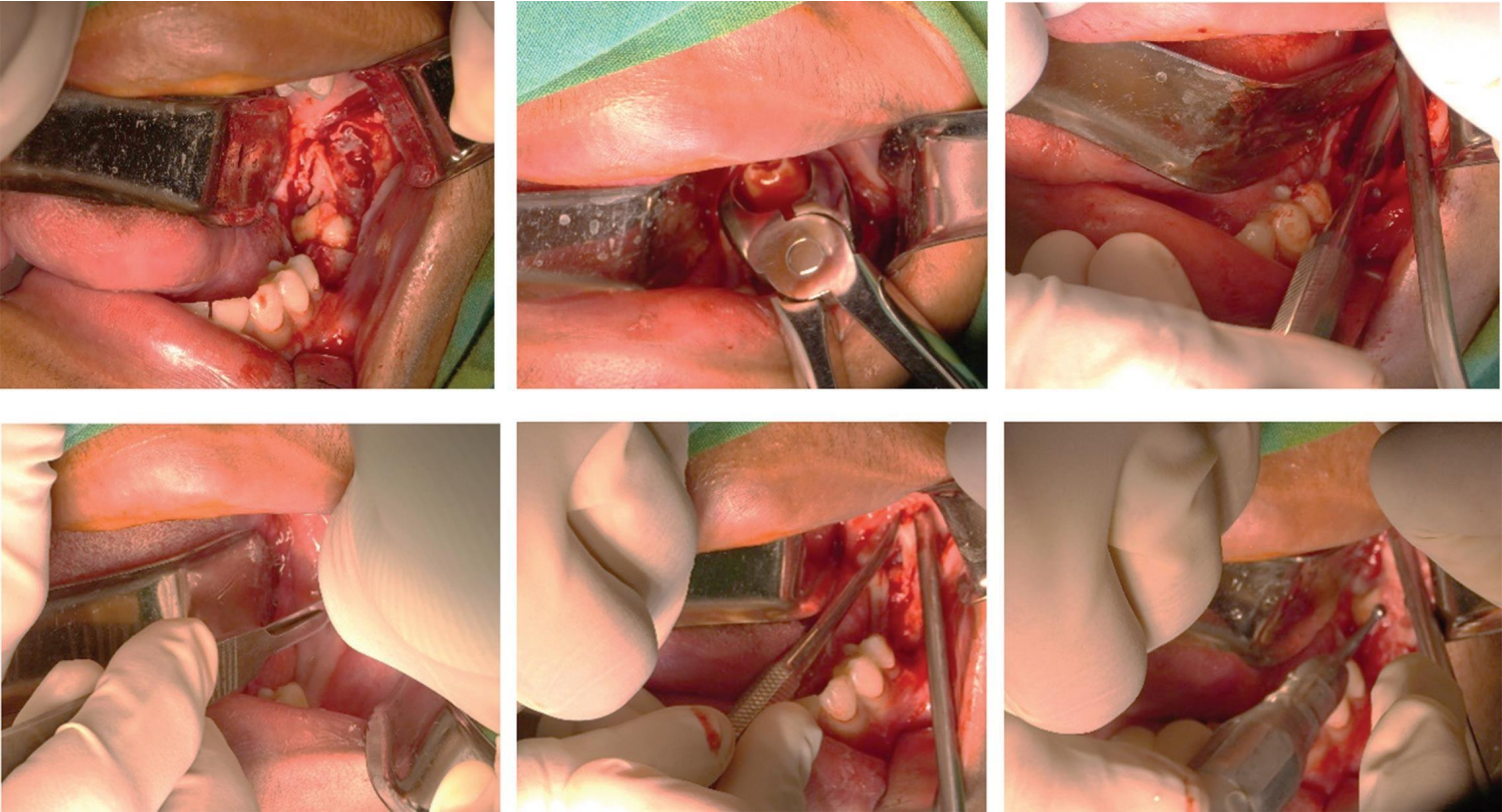
Intraoperative view of Region 38.

**Figure 8:**
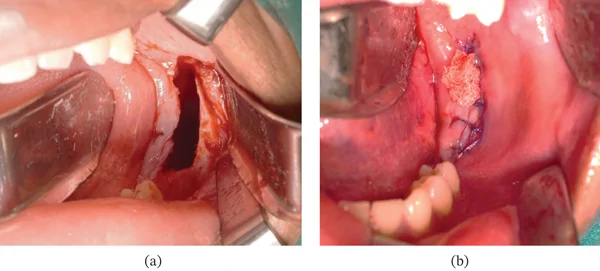
Postoperative view of Region 38. (a) Clinical view of Tooth 38. (b) Application of antibiotic gauze to the surgical site.

After extraoral and intraoral disinfection with povidone‐iodine solution, a vasoconstrictor was infiltrated at the operative site. An incision was made along Teeth 34–38 using a No. 15 blade. Teeth 37 and 38 were extracted. The cyst was separated from the surrounding wall by blunt dissection while preserving the integrity of the cystic lining and the inferior alveolar nerve. Granulation tissue was removed by curettage (Figure 7). The cavity was irrigated alternately with 0.9% sodium chloride and povidone‐iodine solution, followed by gentamicin irrigation for approximately 5 min. Antibiotic gauze was placed in the left mandibular bone defect, and the flap was repositioned and sutured with multiple Vicryl 3‐0 sutures (Figure 8).

**Figure 9:**
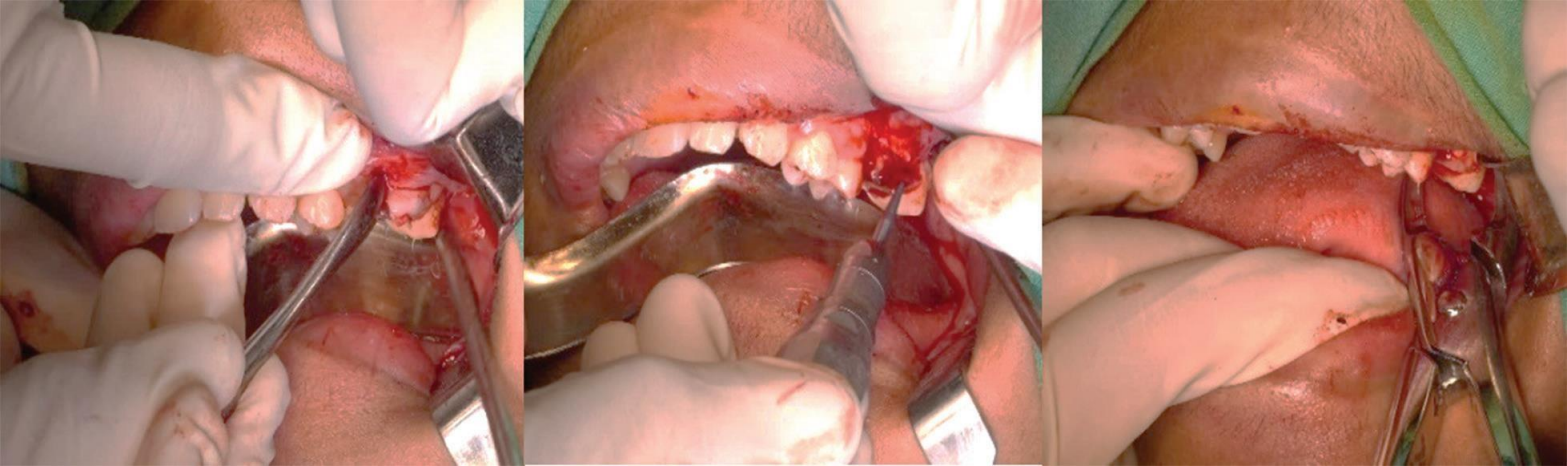
Intraoperative view of Region 28.

**Figure 10:**
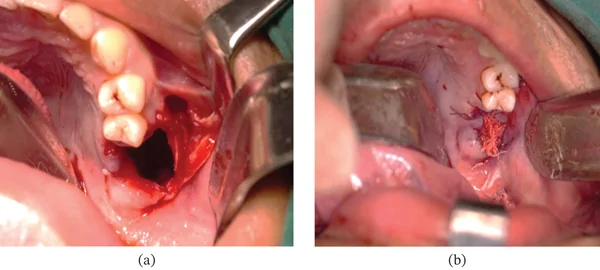
Postoperative view of Region 28. (a) Clinical view of Teeth 27 and 28. (b) Application of antibiotic gauze to the surgical site.

**Figure 11:**
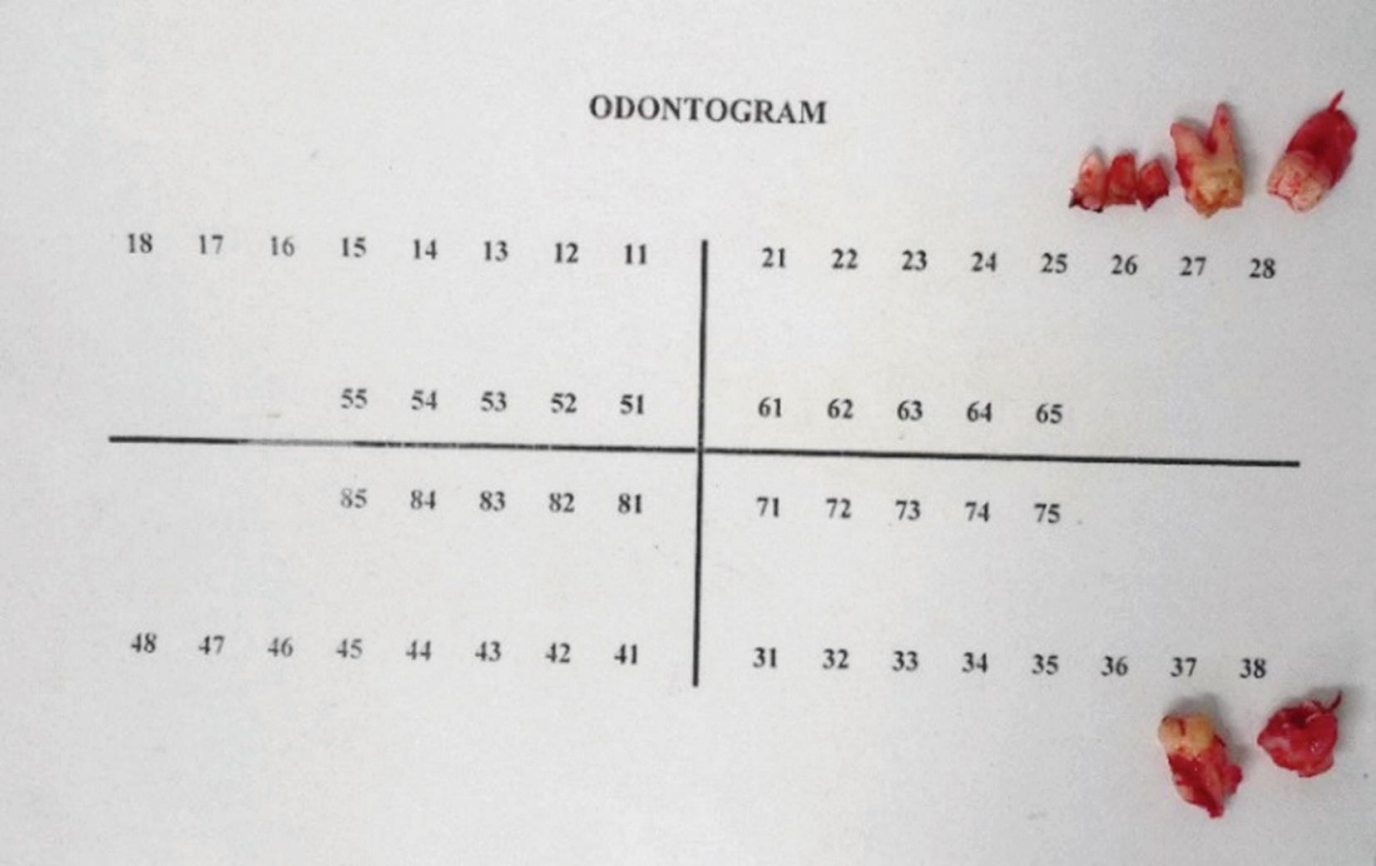
Surgical specimens and extracted teeth from the first operation involving Teeth 37, 38, 26, 27, and 28.

**Figure 12:**
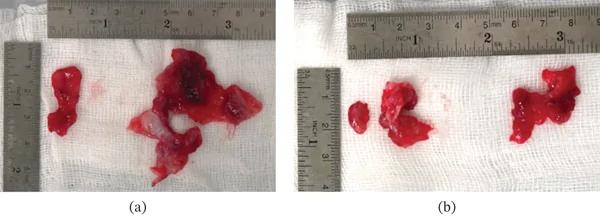
Specimens showing multiple thin cystic soft tissue layers. (a) Left mandibular specimen. (b) Left maxillary specimen.

The left posterior maxillary lesion was managed next. A vasoconstrictor was infiltrated in the vestibular region of Teeth 26–28, followed by a vestibular incision using a No. 15 blade. Teeth 26, 27, and 28 were extracted. The cyst was carefully separated by blunt dissection while maintaining the cystic sac (Figure 9). Granulation tissue was curetted. The cavity was irrigated with 0.9% sodium chloride and povidone‐iodine solution, followed by gentamicin irrigation. Antibiotic gauze was inserted into the bone defect, and multiple Vicryl 3‐0 sutures were placed (Figure 10). The surgical findings and specimens are as seen on Figures 11 and 12. Specimens were stored in 10% formalin and submitted to the anatomical pathology laboratory.

The patient was monitored in hospital until the third postoperative day. Postoperative medication included cefixime, ibuprofen, dexamethasone 0.5 mg tablets, vitamin C, and vitamin B complex. He was advised to maintain oral hygiene through tooth brushing and flossing, to use povidone‐iodine mouthwash 24 h after surgery, to follow a soft diet for approximately 1 week, and to avoid hot foods for 48 h.

**Figure 13:**
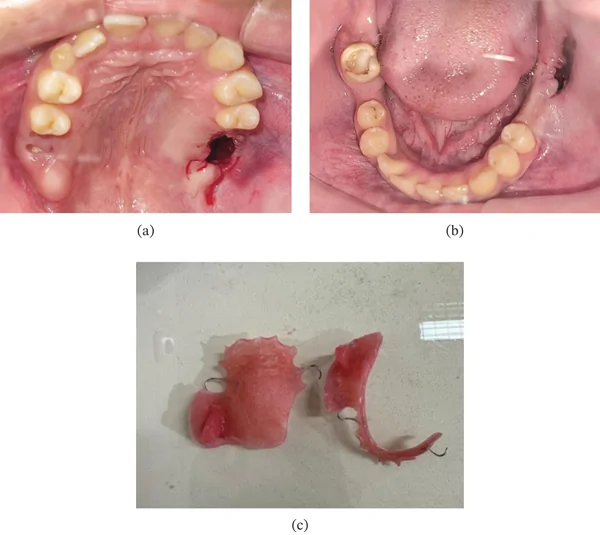
Wound defect after surgery, Day 32. (a) Maxilla. (b) Mandible. (c) Obturator device.

**Figure 14:**
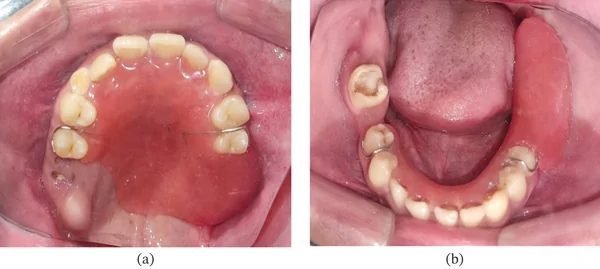
Postinsertion view of the obturator. (a) Maxilla. (b) Mandible.

**Figure 15:**
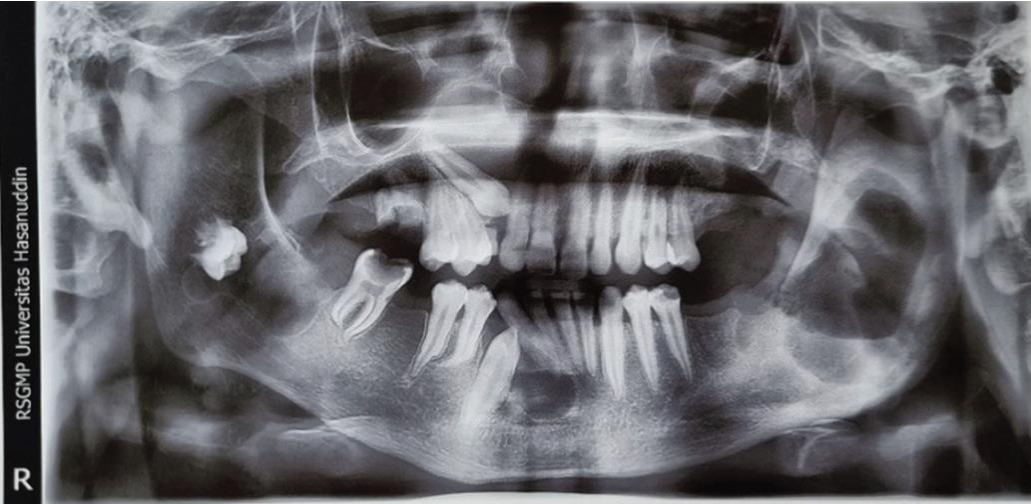
Postoperative panoramic radiographic control at 3 months.

Follow‐up visits were performed 4–10 days after surgery at the Oral and Maxillofacial Surgery Clinic, Dental Hospital of Hasanuddin University. The antibiotic gauze was replaced at each visit. No postoperative complications were observed, and the patient reported no paresthesia, facial edema, or other complaints. On the tenth postoperative day, he was referred to the Prosthodontics Clinic for fabrication of an obturator device (Figure 13). One month after surgery, the obturator was well fitted in the maxilla and mandible, and the patient was able to maintain adequate oral intake (Figure 14). Three months after the first surgery, panoramic radiographic evaluation showed early new bone formation (Figure 15).

**Figure 16:**
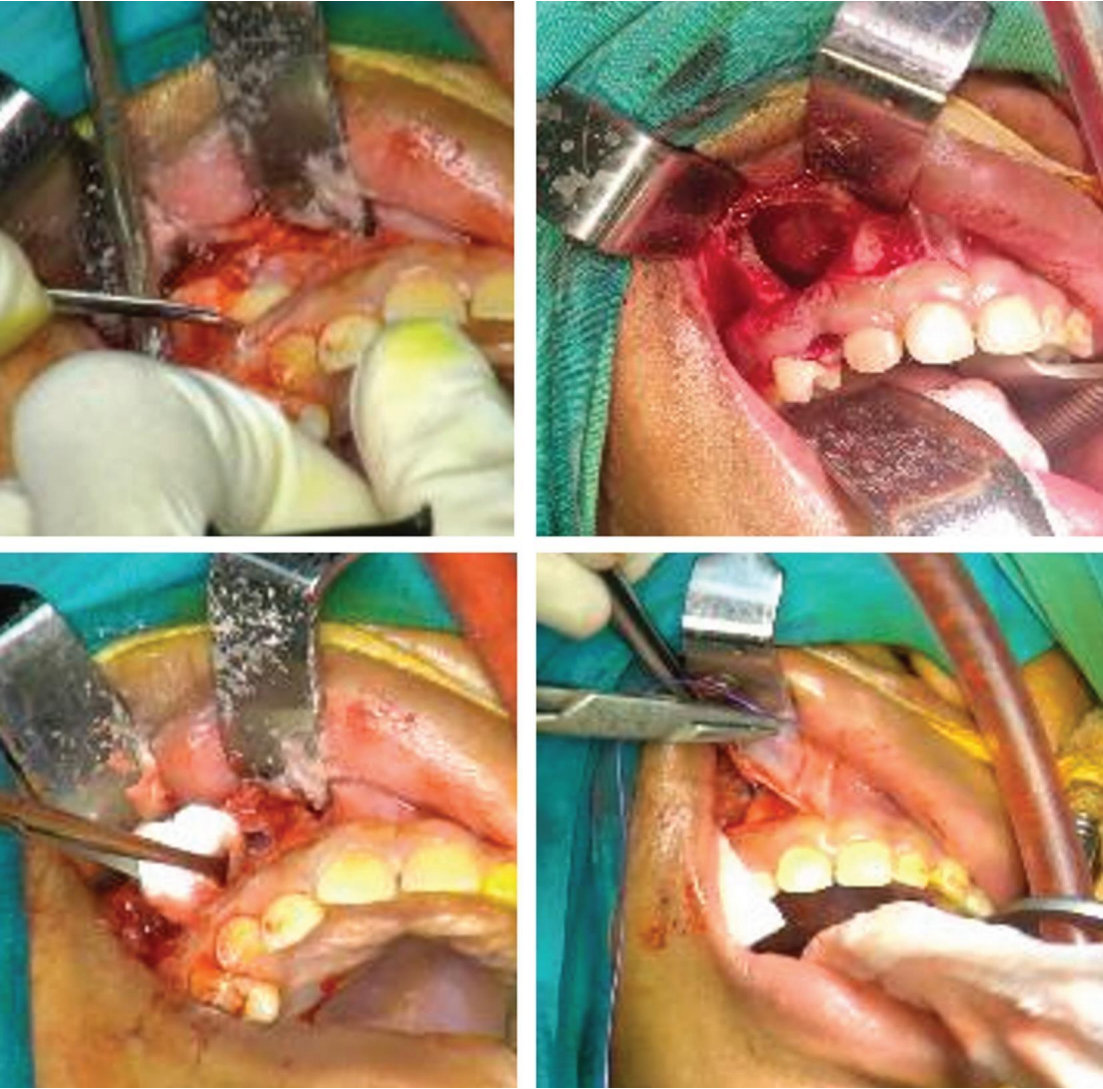
Intraoperative view of Region 13.

**Figure 17:**
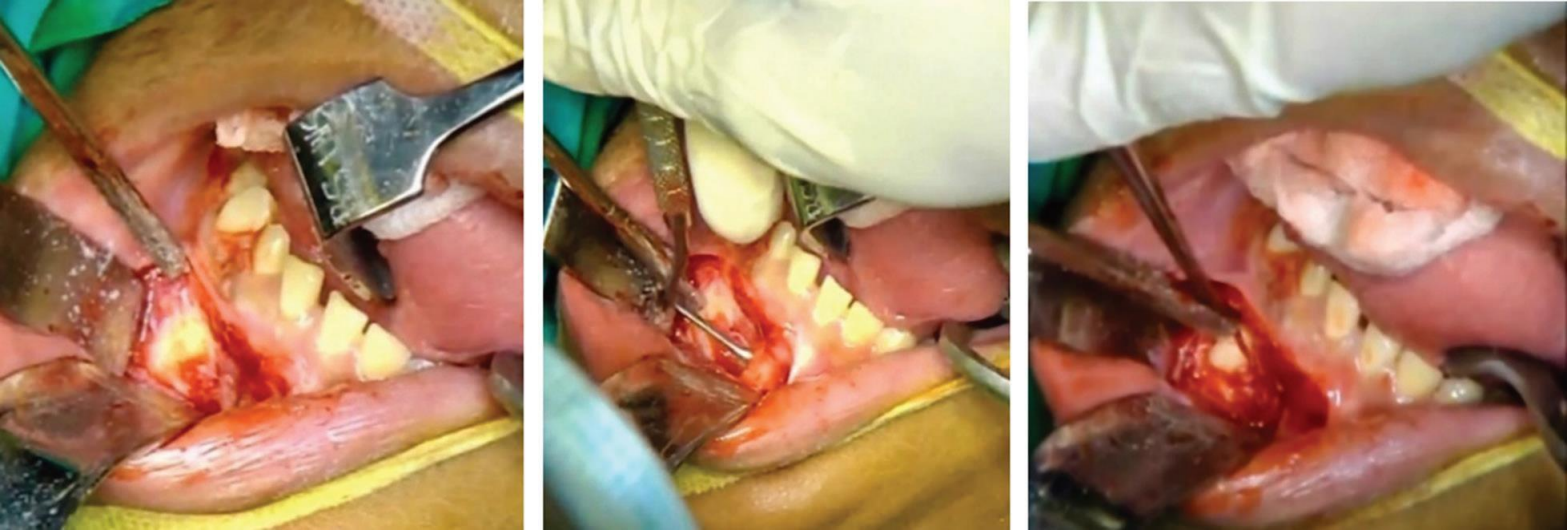
Intraoperative view of Region 43.

**Figure 18:**
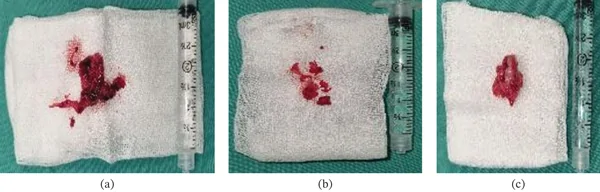
Specimens showing multiple thin cystic soft tissue layers. (a) Region 13. (b) Region 43. (c) Region 48.

**Figure 19:**
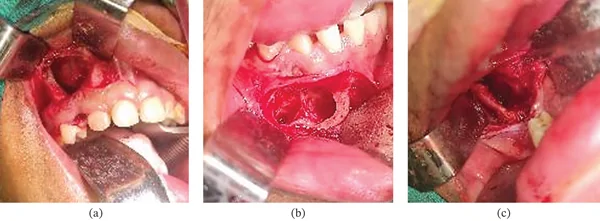
Postoperative views. (a) Region 13. (b) Region 43. (c) Region 48.

**Figure 20:**
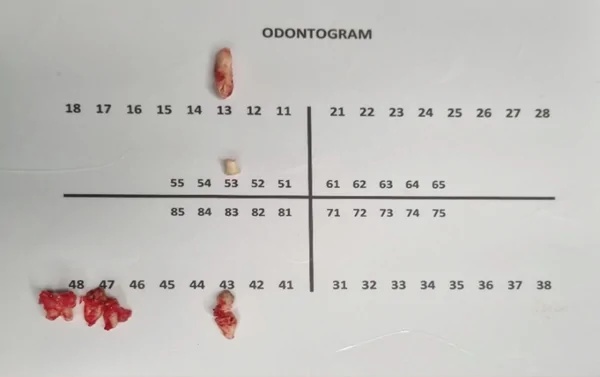
Surgical specimens and extracted teeth from the second operation involving Teeth 13, 43, 47, and 48.

In September 2025, the patient underwent a second enucleation procedure for right maxillary and mandibular lesions using the same operative principles as the first surgery (Figures 16 and 17). The specimens were placed in 10% formalin and submitted for anatomical pathology examination (Figure 18). Antibiotic gauze was inserted into the defects, and the flaps were sutured with multiple Vicryl 3‐0 sutures (Figure 19). The second surgical procedure included extraction of Teeth 13, 43, 47, and 48 (Figure 20).

**Figure 21:**
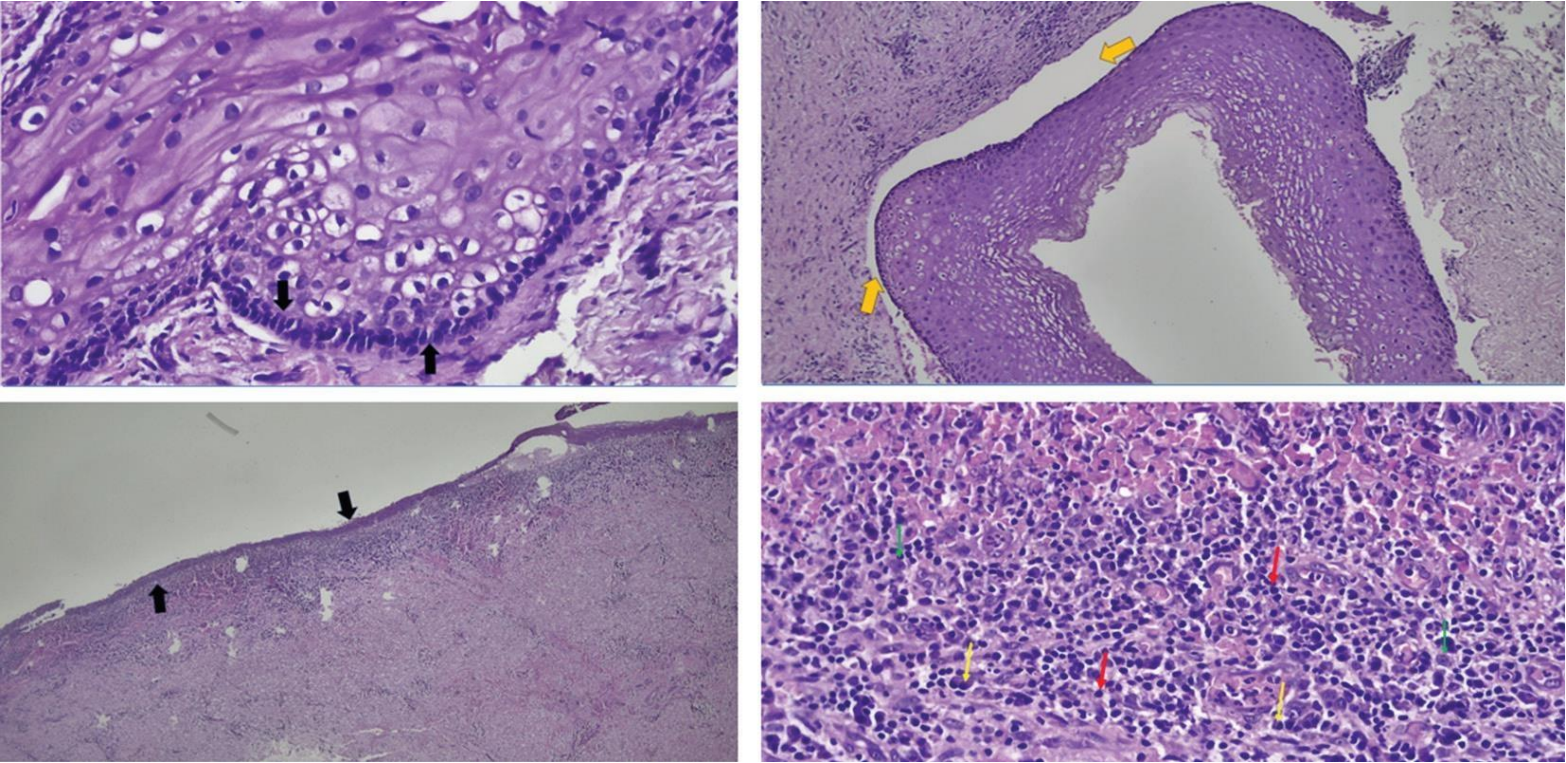
Histopathological features of the odontogenic keratocyst.

Histopathological examination showed cystic structures lined by stratified squamous epithelium with nonatypical nuclei. The basal layer contained hyperchromatic odontogenic cells arranged in a dense cuboidal to columnar pattern. Clefting was present between the epithelium and connective tissue. The subepithelial layer contained inflammatory cells, including lymphocytes, histiocytes, plasma cells, and polymorphonuclear leukocytes (Figure 21). Based on the clinical, radiologic, and histopathologic findings, the final diagnosis was syndromic multiple OKCs of the maxilla and mandible. Six‐month postoperative follow‐up was planned to assess healing, function, and recurrence.

## 3. Patient Perspective

A formal patient‐perspective statement was not obtained. The patient and guardian were informed about the diagnosis, staged treatment plan, need for long‐term follow‐up, and possibility of recurrence.

## 4. Discussion

Gorlin–Goltz syndrome is a rare hereditary disorder in which multiple OKCs may be one of the earliest clinical signs [1]. The syndrome has an estimated incidence of approximately 1 in 50,000–150,000 individuals. Compared with isolated multiple cysts, syndromic multiple OKCs are more commonly associated with systemic skeletal, cutaneous, and neurologic findings (Table 1) [2, 6].

**Table 1:** Clinical manifestations of Gorlin–Goltz syndrome [1].

Category	Manifestations
Skin	Basal cell nevi, basal cell carcinoma, benign dermal cysts and tumors, dermal calcinosis, and palmar or plantar keratosis.
Dental	Multiple odontogenic keratocysts and mild mandibular prognathism.
Bone	Frontal bossing, bifid ribs, spina bifida, kyphoscoliosis, and brachymetacarpalism.
Eye	Hypertelorism, congenital blindness, and internal strabismus.
Neurologic	Dural calcification, developmental delay or intellectual impairment, and medulloblastoma.
Reproductive	Hypogonadism and ovarian tumors.

The molecular basis of Gorlin–Goltz syndrome is linked mainly to pathogenic changes affecting the PTCH gene and the sonic hedgehog signaling pathway. PTCH normally regulates Smoothened activity and thereby influences cellular proliferation and differentiation during development. Altered signaling can promote abnormal odontogenic epithelial proliferation and increase susceptibility to cyst formation and neoplastic changes [5, 7].

The diagnosis is usually supported by the presence of two major criteria or one major criterion with two minor criteria. Major criteria include multiple OKCs, early‐onset basal cell carcinoma, palmar or plantar pits, bilamellar calcification of the falx cerebri, bifid or fused ribs, and a first‐degree relative with the syndrome. Minor criteria include macrocephaly, congenital malformations, skeletal anomalies, radiologic anomalies, medulloblastoma, and ovarian fibroma (Table 2) [8, 9].

**Table 2:** Major and minor diagnostic criteria for Gorlin–Goltz syndrome [8, 9].

Major criteria	Minor criteria
Multiple or single basal cell carcinoma before 20 years of age.	Macrocephaly adjusted for height.
Multiple odontogenic keratocysts.	Congenital malformations, including cleft lip or palate, frontal bossing, and moderate or severe hypertelorism.
Three or more palmar or plantar pits.	Other skeletal anomalies, including Sprengel deformity, marked pectus deformity, and marked syndactyly of the digits.
Bilamellar calcification of the falx cerebri.	Radiologic anomalies, including bridging of the sella turcica, vertebral anomalies such as hemivertebrae, fusion or elongation of vertebral bodies, and modeling defects of the hands or feet.
Bifid, splayed, or fused ribs.	Medulloblastoma.
First‐degree relative with Gorlin–Goltz syndrome.	Ovarian fibroma.

The present patient fulfilled three major criteria: multiple OKCs, bifid ribs, and falx cerebri calcification. He also had craniofacial features suggestive of a minor criterion, including prominent supraorbital ridges. These findings support a syndromic diagnosis rather than isolated multiple OKCs.

Gorlin–Goltz syndrome is often identified in adolescents and young adults. OKCs can be bilateral and may involve both the maxilla and mandible, especially posterior mandibular regions, canine areas, and incisor regions [10, 11]. In this case, multiple cystic lesions involved both jaws and were associated with impacted teeth, which made staged surgical planning necessary.

A cyst is a pathologic cavity that contains fluid or semisolid material and may or may not be lined by epithelium [12]. Multiple jaw cysts may occur with or without a syndrome, but nonsyndromic multiple OKCs are uncommon [13]. Classic OKCs are recognized by their developmental origin, characteristic epithelial lining, and recurrence potential [14]. Radiographically, OKCs usually appear as well‐demarcated unilocular radiolucencies, although multilocular patterns can occur. They may grow extensively with limited expansion and may remain asymptomatic unless secondarily infected [15].

Histologically, OKCs typically show a parakeratinized or orthokeratinized epithelial surface, relatively uniform epithelial thickness, and palisaded basal cells with a tombstone or picket‐fence arrangement. Daughter cysts or satellite cysts may be found in the connective tissue and are associated with recurrence risk. Parakeratinized OKCs are generally considered more aggressive than orthokeratinized lesions, and syndromic OKCs are frequently parakeratinized [4, 9, 14]. In the present case, the epithelial and basal cell features were consistent with OKC, and the syndromic diagnosis was supported by extraoral and radiographic criteria.

Dentigerous cyst should be considered in the differential diagnosis because it may also be associated with impacted teeth. Dentigerous cysts usually arise from reduced enamel epithelium surrounding the crown of an unerupted tooth, grow slowly, and are often asymptomatic [16, 17]. Radiographically, they are typically unilocular radiolucencies attached to the crown of an unerupted tooth and bounded by a corticated margin. A follicular space larger than 5 mm can suggest a dentigerous cyst [18]. Unlike the present case, dentigerous cysts are usually solitary and are lined by nonkeratinized‐stratified squamous epithelium.

Management of OKCs ranges from enucleation and curettage to decompression, marsupialization, adjunctive chemical therapy, and resection in selected cases [2, 3]. The staged approach in this patient was chosen because lesions were present bilaterally in the maxilla and mandible, several teeth were impacted, and the left mandibular lesion posed a higher fracture risk. Long‐term review is essential because recurrence may occur years after treatment, and multiple OKCs may precede other manifestations of Gorlin–Goltz syndrome [9, 19]. Genetic counseling and multidisciplinary evaluation involving oral and maxillofacial surgeons, dermatologists, geneticists, and pathologists are therefore recommended.

This case study has several strengths, including comprehensive clinical, radiographic, and histopathological documentation, as well as detailed reporting of the staged surgical approach and postoperative rehabilitation. The multidisciplinary approach and long‐term evaluation further enhance the validity of the findings. However, its limitations include the single‐case design, which limits generalizability, the absence of formal patient‐perspective data, and relatively short‐term follow‐up. Further studies with larger samples and genetic monitoring are needed to confirm these findings and identify additional risk factors.

## 5. Conclusion

Multiple OKCs in an adolescent patient should alert clinicians to the possibility of Gorlin–Goltz syndrome, especially when jaw lesions are bilateral or associated with skeletal and cranial radiologic findings. In this case, multiple OKCs, bifid ribs, and falx cerebri calcification supported the syndromic diagnosis.

Staged enucleation, careful preservation of vital structures, obturator rehabilitation, and scheduled follow‐up provided satisfactory early clinical outcomes. Long‐term surveillance remains necessary because of the recurrence potential of OKCs and the possibility of additional syndromic manifestations. Genetic evaluation is also recommended to confirm the underlying chromosomal or molecular abnormality and guide family counseling.

## Author Contributions

**Faizal Manrapi:** writing – review & editing, writing – original draft, conceptualization. **Mohammad Gazali:** writing – review & editing, writing – original draft, conceptualization, validation, project administration. **Nurwahida:** writing – review & editing, writing – original draft, conceptualization. **Nisrina Ekayani Nasrun:** writing – review & editing, supervision, project administration.

## Funding

No funding was received for this manuscript.

## Disclosure

All authors have read and approved the final version of the manuscript. Mohammad Gazali, as the corresponding author and manuscript guarantor, had full access to all of the data in this study and takes complete responsibility for the integrity of the data and the accuracy of the data analysis. The authors checked the cited references for retraction notices and published corrections using PubMed, publisher websites, and the Retraction Watch database before resubmission. No cited reference was identified as retracted, and no published correction requiring reinterpretation of the cited evidence was identified at the time of resubmission.

## Ethics Statement

Ethical approval was not required for this single anonymized case report because it describes routine clinical care and does not include identifiable patient information. Written informed consent for treatment and publication was obtained from the patient and guardian. This report was prepared according to the CARE guidelines and in accordance with the principles of the Declaration of Helsinki.

## Consent

The patient and guardian provided written informed consent for publication of clinical details, intraoral photographs, radiographic images, and treatment information in this case report.

## Conflicts of Interest

The authors declare no conflicts of interest.

## Data Availability

The datasets generated and/or analyzed during the current study are available from the corresponding author on reasonable request.
